# Sevoflurane increases locomotion activity in mice

**DOI:** 10.1371/journal.pone.0206649

**Published:** 2019-05-21

**Authors:** Hoai T. Ton, Lei Yang, Zhongcong Xie

**Affiliations:** 1 Geriatric Anesthesia Research Unit, Department of Anesthesia, Critical Care and Pain Medicine, Massachusetts General Hospital and Harvard Medical School, Charlestown, MA, United States of America; 2 Department of Biology, Vinh University, Vinh City, Nghe An, Vietnam; 3 Department of Anesthesia, Ruijing Hospital, Shanghai Jiaotong University, Shanghai, China; Jinling Clinical Medical College of Nanjing Medical University, CHINA

## Abstract

Clinical observations show emergence of agitation and hyperactivity during the anesthesia induction and/or recovery period post-anesthesia. However, an animal model to illustrate this clinical phenomenon has not yet been established. We therefore set out to investigate whether sevoflurane, a commonly used anesthetic, could alter locomotion in mice during the anesthesia induction and recovery period post-anesthesia. The activity of the mice was recorded 5 minutes before, during (for 30 minutes), and 40 minutes after the administration of the anesthetic sevoflurane [1-, 1.5- and 2-fold minimum alveolar concentration] at 37^0^ C. The total walking distance and velocity of movement were measured and quantified as the indexes of locomotion. We found that the anesthetic sevoflurane increased the locomotion of the mice during the induction period of the anesthesia. During the recovery phase after anesthesia, the mice exhibited increased locomotion for a short period of time (about 5 minutes) and then displayed a sharp decrease in mobility for up to 60 minutes following the end of anesthesia administration. The anesthetic sevoflurane did not significantly alter the food intake and body weight of the mice. Furthermore, we found that Alzheimer’s disease transgenic mice exhibited a greater degree of sevoflurane-induced hyperactivity than the wild-type mice did. Our results showed that inhalation of the anesthetic sevoflurane induced an acute hyperactivity in mice, particularly among Alzheimer’s disease transgenic mice. These findings from the pilot studies have established an animal model to promote further studies into postoperative emergence agitation, hyperactivity and the underlying mechanisms into these conditions.

## Introduction

Sevoflurane is one of the most commonly used general anesthetics. Despite its merits, several clinical studies have reported the emergence agitation and hyperactivity that emerges after the administration of sevoflurane, particularly for pediatric patients who exhibit the incidence of emergence agitation up to 80%[1–3]. Moreover, the transient state of marked irritation and disassociation was observed during the transition from the state of unconsciousness to complete wakefulness or following the discontinuation of anesthesia in humans[3,4]. Specifically, clinical investigations have shown that sevoflurane can induce hyperactivity during mask induction, even causing body movements, epileptiform electroencephalographic activity, seizure-like movements, and actual seizures[1,2,4–7]. Such events pose a risk of injury and potential postoperative complications. However, the underlying mechanisms and targeted intervention(s) of such clinical observations remain unknown.

Recent *in vivo* studies have showed that, particularly during the early stages of Alzheimer Disease (AD), neuronal circuits are hyperactive instead of hypoactive[8–11], which could lead to key changes in AD[12]. Interestingly, sevoflurane has been shown to promote AD-associated apoptosis, Tau protein phosphorylation^10-11^, and β-amyloid protein accumulation (Aβ)^9^, the major pathological hallmarks of AD neuropathogenesis, in cultured cells and in animals. Previous studies have assessed the effects of sevoflurane on neurotoxicity in wild-type (WT) and AD transgenic (Tg) mice[13,14]. However, the acute behavioral change in locomotion following the administration of the anesthetic sevoflurane has not been fully elucidated. Therefore, the objective of the present study was to establish a system to assess the acute effects of sevoflurane on locomotion in mice. The hypothesis in these current studies was that the anesthetic sevoflurane increased locomotion in mice. We further compared the effects of the sevoflurane-induced changes between the WT and AD Tg mice. We also examined the effect of sevoflurane on food intake and body weight from days 1 to 4 post-anesthesia.

## Methods

### Animals

Animal testing was conducted in accordance with the National Institute of Health guidelines and regulations. The Massachusetts General Hospital Standing Committee on the Use of Animals in Research and Teaching (Boston, Massachusetts) has approved the animal protocol (Protocol number: 2006N000219). Animals were kept in a temperature-controlled (22–23°C) room under a 12-h light/dark period (light on at 7:00 AM); standard mouse food and water were available ad libitum. Housing was provided along with appropriate tactile, olfactory, visual, and auditory stimuli. Every effort was made to minimize the number of mice that were used in experiments. The study was performed on WT C57BL/6J and AD Tg 9 months-old female mice (Jackson Lab, Bar Harbor, ME). The AD Tg mice were purchased from the Jackson Lab (B6SJL Tg APPSwFlLon,PSEN1*M146L*L286V)6799Vas/Mmjax; Stock Number: 006554), and maintained in our own laboratory. Standard genotyping techniques were used to confirm the conditions of the AD Tg mice. All efforts were made to minimize suffering of the animals.

### Anesthesia in mice

Prior to carrying out the experiments, we familiarized the mice to the transparent gas-tight plastic chamber (20 x 20 x 15 cm) by placing each of them, without anesthesia, into the chamber for 10 minutes once per day for 3 consecutive days. The experiments were conducted between 9:00 a.m. and 12:00 p.m. Then after, the mice were randomly assigned into the anesthesia group or control group. In the anesthesia group, the mice were placed into the chamber and exposed to sevoflurane for 30 minutes (for locomotion activity assessment), for 4-hours (for food intake and gain weight assessment) or not at all (the control condition). We used air containing 40% O2/60% N2 as a carrier and the total gas flow was 2 L/min. During the exposure to anesthesia, the mice were kept warm on a plate heated to 37°C with a white colored background. The control group was exposed to the air containing 40% oxygen and balanced with nitrogen with an equal rate of flow in a chamber which was similar to the anesthesia chamber. The Dash 4000 monitor (GE Medical Systems information technologies INC., Wisconsion, USA) was used to determine the concentrations of sevoflurane. The fate of all experimental animals is being killed at the end of each measurement by 100% CO_2_ to prevent any extension of stress or pain.

### Locomotion measurement

While their horizontal ambulatory activity was measured with the aid of a video-tracking system, the animals were allowed to move freely in the transparent gas-tight plastic chamber. This allowed us to measure all the required parameters offline under simple blind conditions using the ImageJ software. Mice activity was recorded before (5 minutes in air as baseline tracks), during (30 minutes) and after the sevoflurane anesthesia (40-minutes recovery) exposure. The movement traces were then analyzed using the Multi Tracker Plugin function in the Image J software. Here, we used 1-, 1.5- and 2-fold minimum alveolar concentrations (MAC), which correspond to 2, 3, and 4% of sevoflurane respectively at 37^0^ C[1,3]^,^[15]. The total walking distance (in cm) and velocity of movement (in cm/min; maximal value in induction and mean value in baseline and recovery) were measured and quantified as indexes of hyperactivity. A student’s t-test was used to determine the difference between the mice in the anesthesia and control condition.

Following the experimental session, the mice were carefully removed from the chamber, and returned to their home cage. The test equipment was cleaned with 50% ethanol solution and dried in between subjects in order to avoid olfactory cuing. All of the mice were used only once.

### Food intake and body weight

The food intake and body weight were measured after daily anesthesia exposure between the hours of 9:00 a.m. and 12:00 p.m. Food and body weight were weighed from days 1 to 4 post-anesthesia. Food intake was calculated as the amount of food removed from the feeding bowl.

### Statistical analysis

Data are presented as mean values with the standard error of the mean. The number of samples varied from 14 to 16 in each group for the measurement of food intake and weight changes. The number of mice used for measuring locomotion ranged from 4 to 6 in each group. The power determination from the pilot studies suggested that 4 to 6 mice in each group would be sufficient to illustrate the difference in locomotion between the mice in the sevoflurane anesthesia and the mice in the control condition. Data analysis was conducted using the Origin 8.0 software (OriginLab Corporation, Northampton, MA). A student’s t-test was used to determine the difference between the mice in the anesthesia and control condition in terms of the walking distance and velocity. A one-way ANOVA was used to analyze the difference in food intake and the weight change between mice in the control group and mice in the anesthesia group. Statistical significance was determined as p<0.05.

## Results

### Sevoflurane induced hyperactivity in the mice

**Fig 1:**
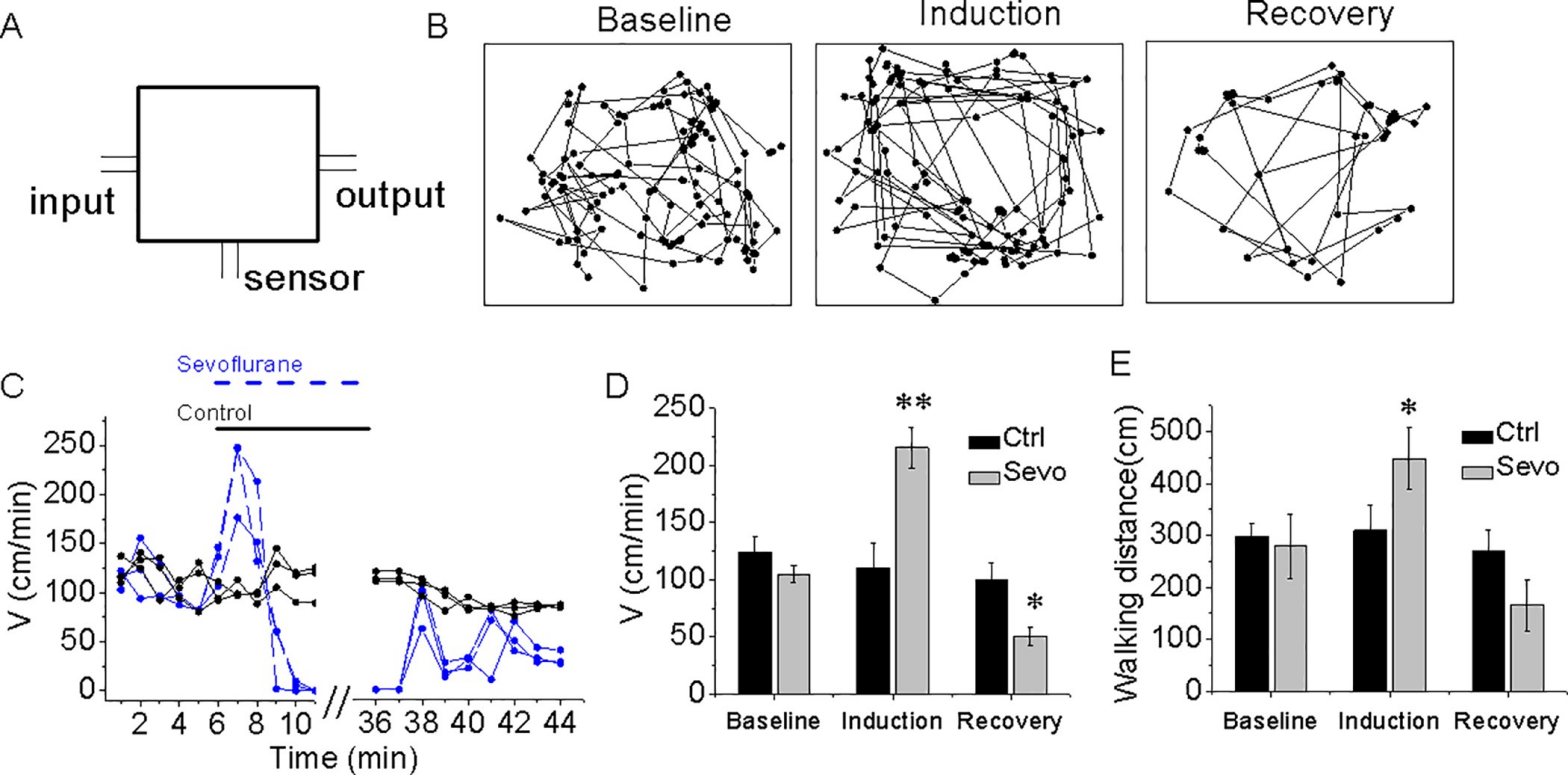
Sevoflurane increases locomotion in mice. (A) Diagram of the chamber used to treat the mice with sevoflurane (2%) in N_2_/O_2_ and access locomotion. Control mice received only N_2_/O_2_. (B) Representative traces demonstrating the position of the mouse in the activity chamber before, during, and after administration of 2% sevoflurane anesthesia (C) the time-course of mean velocity of 3 mice per group during a single exposure to the sevoflurane or control condition. (D & E) Summary of mean velocity and walking distance for 2 minutes during each period of the baseline, anesthesia induction and anesthesia recovery of the sevoflurane-treated mice (green bar) and control condition mice (black bar). Data shown as mean and SEM, n = 4 and 6 for control and sevoflurane-treated group, respectively.

Sevoflurane has been reported to induce hyperactivity during the time of anesthesia induction and post-anesthesia recovery period in patients[2]. To assess whether sevoflurane would induce hyperactivity in animals, we tracked the horizontal ambulatory activity before and during the induction of the anesthetic sevoflurane as well as after the sevoflurane administration to each of the mice. As shown in Fig 1, all of the mice tested had similar baseline levels of velocity ranging from 100 to 130 cm/minute. However, the anesthetic sevoflurane markedly increased the locomotion during the time of the anesthesia induction compared to that of the control condition as evidenced by the 76% increase in the velocity (194.4 ± 47.2 versus 110.3 ± 21.6 cm/min) and 45% increase in the walking distance (447.9 ± 58.3 versus 309.3 ± 49.3 cm). However, during the recovery period following the anesthesia, there were short periods (within 5 minutes) of increased locomotion, which were followed by decreases in locomotion during the next 20 minutes in terms of velocity (45.9 ± 14.4 versus 99.3 ± 15.5 cm/min) and walking distance (165.9 ± 49.3 versus 270.9 ± 39.3 cm). The mice activity level recovered to the baseline level 60 minutes after the end of the sevoflurane anesthesia exposure. We noted that there was a small individual difference in the locomotion among each of the mice. However, the conclusion was not altered by such variations in locomotion.

**Fig 2:**
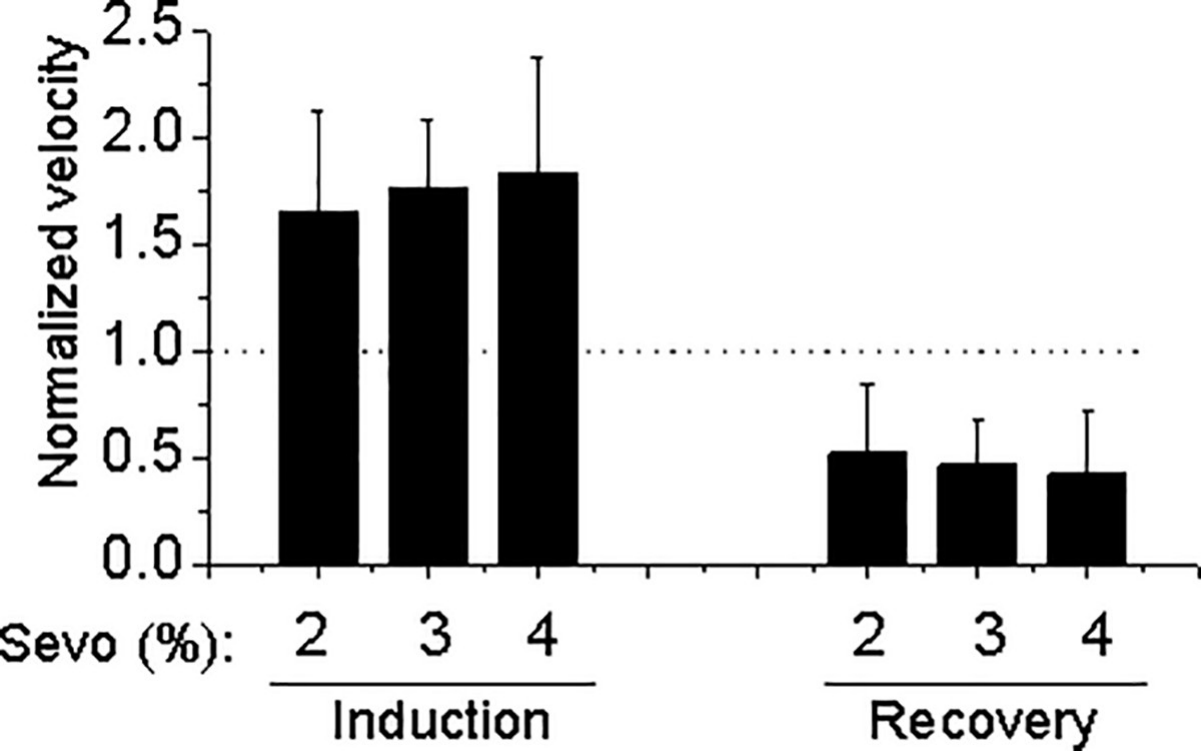
Ratio of mean speed induced by sevoflurane at different concentrations of sevoflurane and baseline. Normalized velocity shown as mean ± SEM using 4–6 mice in each group. Note that the sevoflurane-induced moving pattern (hyperactivity during induction and hypoactivity during recovery) in the mouse model was observed at all tested concentrations ranging from 2–4% sevoflurane with undetectable statistical significance among the different concentrations.

Next, we tested the effect of sevoflurane on the locomotion at different concentrations ranging from 2, 3 to 4% (1-, 1.5- and 2-fold MAC). Fig 2 shows that sevoflurane increased locomotion at all of the concentration levels tested. However, we did not observe a statistically significant difference among the effects different concentrations of sevoflurane had on the hyperactivity in the anesthesia induction phase or recovery phase post-anesthesia (Fig 2). We noted that the higher concentrations of sevoflurane the mice received, the faster they fell asleep, shortening the duration of their hyperactivity and thus potentially preventing the possible concentration-dependent effect of sevoflurane on hyperactivity from being observed during the induction of the anesthesia.

### The sevoflurane-induced hyperactivity was greater in AD Tg mice

Recent *in vivo* evidence from mouse models and human patients have indicated that, particularly during the early stages of AD, neuronal circuits are hyperactive instead of hypoactive[8–11], which could lead to key complications in AD[12]. Therefore, we questioned how sevoflurane-induced movements in AD Tg mice.

**Fig 3:**
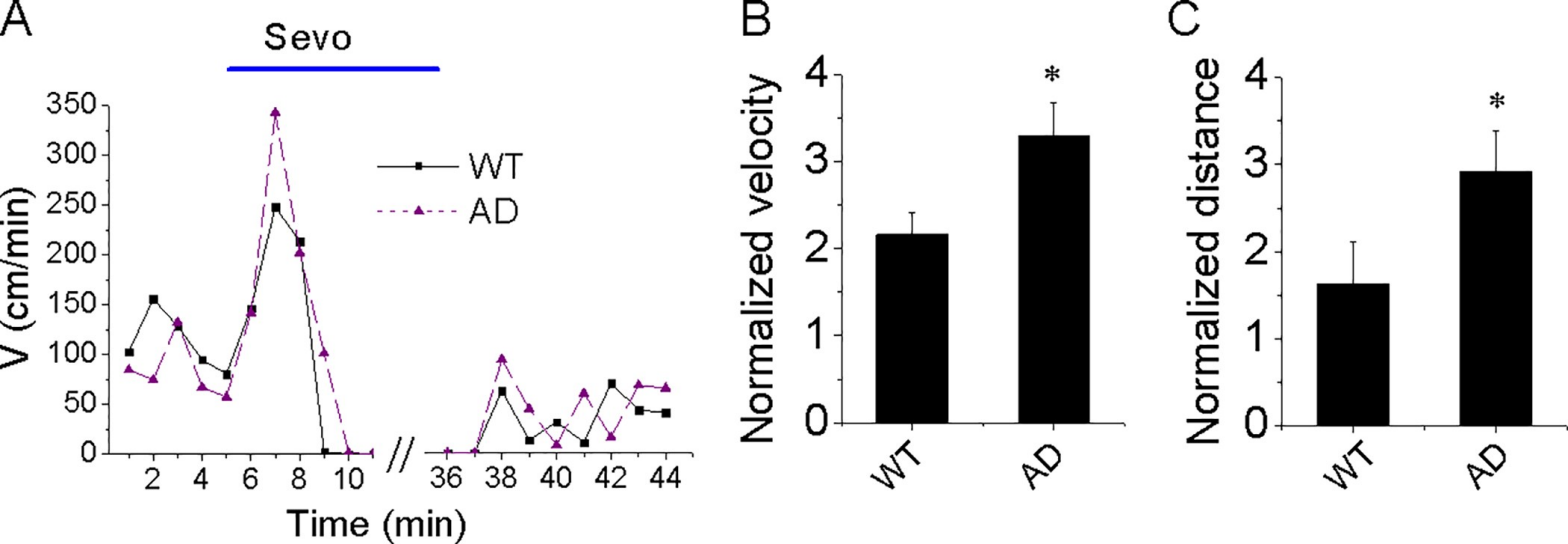
Sevoflurane-induced hyperactivity in AD Tg mice compared with WT mice. (A) The time-course of movement velocity in the activity chamber before and after the exposure to sevoflurane in the WT and AD Tg mice; (B&C) Summary of normalized velocity and walking distance in the induction period for each mouse type. Data shown as mean and SEM, n = 7 and 9 mice in WT and AD Tg group, respectively. Note that the sevoflurane-induced hyperactivity is increased in the AD Tg mice.

To answer this question, we performed further experiments using AD Tg mice. Fig 3A shows that the sevoflurane-induced hyperactivity in AD Tg mice was greater than that of the WT mice (342.6 ± 42.4 cm/min for AD Tg mice versus 247.9 ± 36.8 cm/min for WT mice). Given the findings that the baseline velocity of mice was different (103.8 for AD Tg mice versus 119.04 cm/min for WT mice), we calculated the ratio of locomotion, which consisted of the 2% sevoflurane to baseline levels in each mouse, and graphed out the normalized parameters to show the results of locomotion in AD Tg mice and WT mice (Fig 3B and 3C). We found that the ratios of velocity and walking distance were still significantly higher in AD Tg mice compared to those in the WT mice (velocity: 3.3 ± 0.4 versus 2.1 ± 0.26; walking distance = 2.9 ± 0.5 versus 1.6 ± 0.5, p < 0.05, student’s t-test). Taken together, these data showed that the exposure to sevoflurane increased the locomotion in WT and AD Tg mice, with a greater increase in the latter group.

### Sevoflurane did not change food intake and body weight of the mice

**Fig 4:**
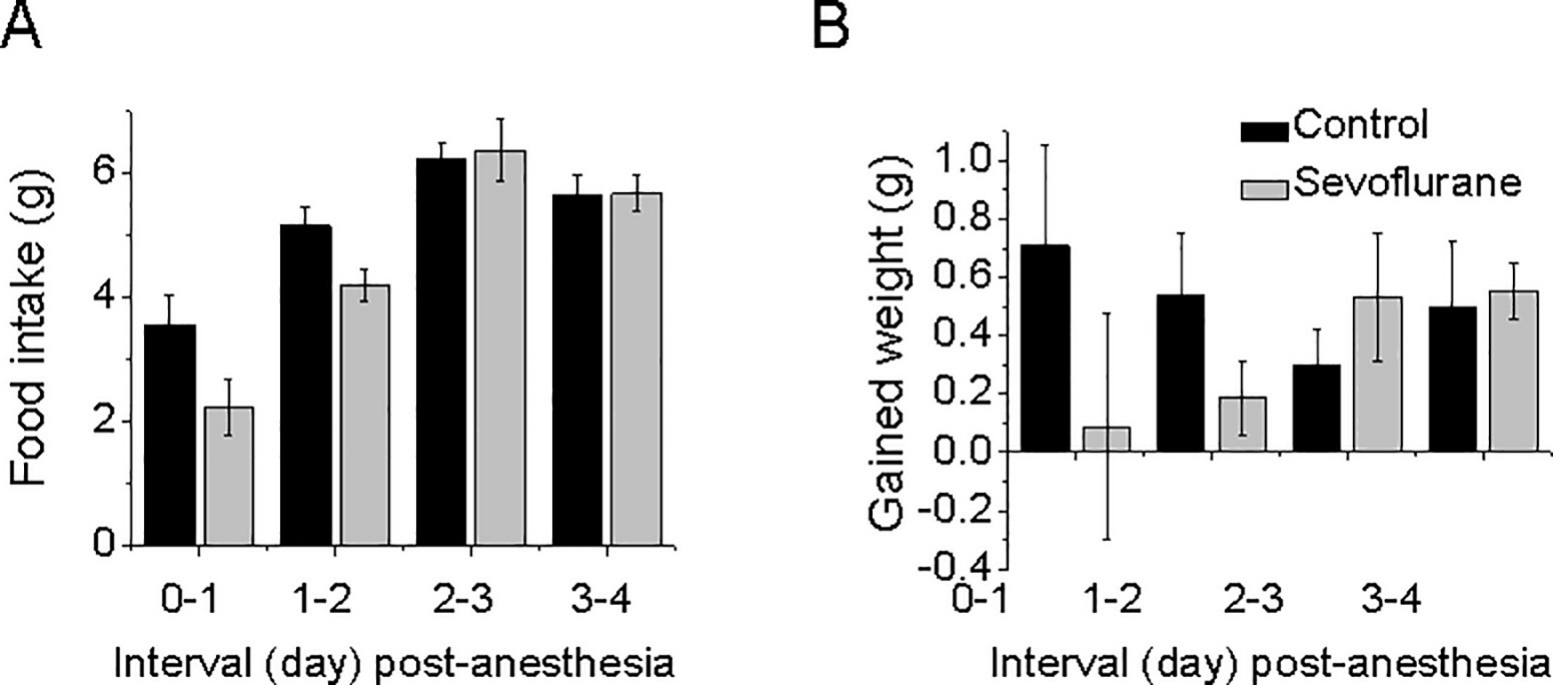
Food intake and weight gains after the 4-hour treatments of 3% sevoflurane. Data are shown as mean ± SEM with n = 14 and 15 mice in control and sevoflurane-treated group, respectively. Note that there is statistically significant difference in food intake (A) and that weight gained(B) from 1 to 4 days after the administration of sevoflurane was not observed between the mice in the sevoflurane-treated group and the mice in the control group.

Food intake and body weight involve a complex integration of hormonal, neuronal, physiologic, and metabolic factors. We observed that sevoflurane has an acute effect on locomotion (during the induction and recovery period but all the indexes returned to normal one hour after the treatment). We questioned whether sevoflurane could have long-lasting effects on the physiology and metabolism of the mice by measuring their food intake and body weight from days 1 to 4 post-anesthesia. In a separate group of animals, we examined the effects of anesthesia with 3% sevoflurane, which was administered for 4 hours, had on food intake and daily body weight in each of the mice. Fig 4 shows that the food intake and body weight of the mice in anesthesia-treatment group were not statistically significant as compared to those observed in the mice in the control group from days 1 to 4 post-anesthesia. These data demonstrate that the anesthetic sevoflurane did not significantly change the food intake and body weight of the mice.

## Discussion

By measuring the velocity and walking distance at baseline levels, we examined the locomotion of the mice under the sevoflurane-induced anesthesia. The data showed that sevoflurane, at clinically-relevant concentrations, induced hyperactivity during the anesthesia induction phase and hypoactivity during the recovery phase post-anesthesia in the WT mice. Moreover, AD Tg mice showed greater hyperactivity following the administration of the anesthetic sevoflurane.

The clinical observation shows that several general anesthetics, especially sevoflurane, induce emergence agitation and hyperactivity in patients [10,12]. It has been reported that the emergence agitation is characterized as hyperactivity, which includes confusion without recognition and eye contact with the surrounding environment as well as increased muscular tension[2]. We therefore assessed the effects of sevoflurane on hyperactivity in mice. However, pending further investigation, whether or not sevoflurane-induced hyperactivity observed in mice has relevance with such clinical observation is still not clear. Moreover, previous studies have showed that sevoflurane at 1% had the ability to activate hippocampal CA3 kainate receptors to induce neuronal hyperactivity, which was measured by electrophysiology, during the anesthesia induction and recovery period post-anesthesia [16]. Whether or not the sevoflurane-induced activation of the hippocampal CA3 kainate receptor contributes to the sevoflurane-induced hyperactivity, however, remains unknown. Therefore, future studies to test this hypothesis are warranted.

Moreover, the data showed that the sevoflurane-induced hyperactivity in AD Tg mice was greater than that observed in the WT mice. Hyperactivity has been reported in patients with AD, which is associated with functional and structural alterations in a distributed network of brain regions supporting memory and other cognitive domains[17]. Evidence that brain hyperactivity occurs in early or pre-symptomatic AD has also been reported. Bookheimer et al., for example, noted that middle aged and elderly people who were carriers of the APOE epsilon4 allele, the genetic risk factor of AD, showed abnormally high activation in the hippocampus and other memory-related brain regions that are typically affected by AD during memory tasks[18]. Putcha et al. found hyperactivity in the hippocampus among patients with mild cognitive impairment[19–22]. Thus, the paradox is that higher levels of hippocampal activity may drive AD disease progress and may be early indicators of AD-related neurodegeneration[11,23]. Our data showed that sevoflurane induced greater hyperactivity in AD Tg mice than in WT mice, which suggest that sevoflurane may induce a greater hyperactivity in AD patients. However, the exact the mechanism and the clinical relevance of such findings need to be further investigated.

Our study has several limitations. First, we did not demonstrate the concentration-dependent effects in the sevoflurane-induced hyperactivities. The exact reason is not clear at the present time. Our anesthetic delivery system did not allow the gas to immediately reach the expected concentrations (i.e. 2, 3 or 4%). Therefore, there was a time of delay (~ 40s) which could have prevented the observation of the possible concentration-dependent effect of the sevoflurane-induced hyperactivity. Secondly, the data were obtained in mice, and the clinical relevance of the sevoflurane-induced hyperactivity in the mice remains to be determined.

In conclusion, we have provided the initial evidence that inhalation of the anesthetic sevoflurane induced an acute hyperactivity in the mice, particularly in the AD Tg mice. This novel observation of sevoflurane-induced hyperactivity would be helpful in the design of an animal model for future studies that looks at emergence agitation and postoperative behavioral changes. Ultimately, these studies may promote further research into the mechanisms of anesthesia action and anesthesia neurotoxicity.

## Supporting information

S1 File Supporting data. Raw data of all experiments showing in the figures.
(XLSX)
